# Food allergy and anaphylaxis – 2039. Bioinformatics evaluation of new proteins in genetically engineered organisms and novel foods for potential risks of food allergy and celiac elicitation

**DOI:** 10.1186/1939-4551-6-S1-P124

**Published:** 2013-04-23

**Authors:** Richard Goodman, Plaimein Amnuaycheewa, John Wise, Steve L Taylor

**Affiliations:** 1 Food Allergy Research and Resource Program, University of Nebraska, Lincoln, USA; 2 Food Science & Technology, Food Allergy Research and Resource Program, University of Nebraska-Lincoln, USA

## Background

Proteins introduced into foods through genetic engineering must be evaluated for potential risks of food allergy (FA) or if derived from grain, potential elicitation of celiac disease (CD). The evaluations are based on Codex Alimentarius guidelines (2003). Novel food ingredients may also be evaluated. Amino acid sequence comparisons are recommended to identify proteins that should be screened further by serum IgE tests for food allergy (FA) or T cell proliferation for potential CD eliciting proteinsprior to use in foods.

## Methods

The www.allergenonline.org database was established in 2004 and updated annually. Criteria were defined by a panel of recognized allergy experts who also review updates annually based on published evidence the proteins are allergenic or are from an allergenic source and bind IgE specifically allergic individuals. Users enter an amino acid sequence for comparison against the database. The new celiac database was constructed by reviewing major publications of native, mutaedor deamidated peptides derived from glutens and suggested as elicitors of CD. Both are available at no cost for public use.

## Results

Version 12 of the allergen database (February 2012) includes 1603 sequences from 603 taxonomic-protein groups associated with IgE mediated allergy. References are provided for each group and sequences can be searched for matches exceeding regulatory criteria. The new experimental celiac database was constructed and released in February 2012 and includes 1016 published peptide sequences from 68 glutens of wheat, barley, rye or oats that are associated with celiac disease. Publications were evaluated regarding reported T cell proliferation from PBMC or clones from MHC Class II DQ 2.5 or DQ 8 restricted CD subjects. Criteria for inclusion of sequences in both databases and appropriate uses are described online. Criteria, simple to follow sequence comparison instructions and interpretations are illustrated.

## Conclusions

The two databases provide efficient and simple tools to evaluate candidate food proteins that might pose a risk of elicitingFA symptoms or CD in affected individuals. Proteins that do not exceed criteria have a small likelihood of elicitingFA or CD in sensitized consumers. Proteins that exceed criteria should be evaluated further or not used in new food sources.

